# Copper-Doped Biphasic Calcium Phosphate Powders: Dopant Release, Cytotoxicity and Antibacterial Properties

**DOI:** 10.3390/ma14092393

**Published:** 2021-05-04

**Authors:** Aurélie Jacobs, Guillaume Renaudin, Nicolas Charbonnel, Jean-Marie Nedelec, Christiane Forestier, Stéphane Descamps

**Affiliations:** 1 Université Clermont Auvergne, Clermont Auvergne INP, CNRS, ICCF, F-63000 Clermont-Ferrand, France; aurel.jacobs@gmail.com (A.J.); jean-marie.nedelec@sigma-clermont.fr (J.-M.N.); 2 Université Clermont Auvergne, CNRS, Laboratoire Microorganismes: Genome et Environnement, F-63000 Clermont-Ferrand, France; nicolas.charbonnel@uca.fr (N.C.); christiane.forestier@uca.fr (C.F.); 3 Université Clermont Auvergne, Clermont Auvergne INP, CNRS, CHU Clermont, ICCF, F-63000 Clermont-Ferrand, France; s_descamps@chu-clermontferrand.fr

**Keywords:** copper-doping, biomaterials, cytotoxicity, antibacterial effect, culture medium

## Abstract

Cytotoxicity and antibacterial properties associated with the dopant release of Cu-doped Biphasic Calcium Phosphate (BCP) powders, mainly composed of hydroxyapatite mixed with β-tricalcium phosphate powders, were investigated. Twelve BCP ceramics were synthesized at three different sintering temperatures (600 °C, 900 °C and 1200 °C) and four copper doping rates (*x* = 0.0, 0.05, 0.10 and 0.20, corresponding to the stoichiometric amount of copper in Ca_10_Cu*_x_*(PO_4_)_6_(OH)_2-2*x*_O_2*x*_). Cytotoxicity assessments of Cu-doped BCP powders, using MTT assay with human-Mesenchymal Stem Cells (h-MSCs), indicated no cytotoxicity and the release of less than 12 ppm of copper into the biological medium. The antibacterial activity of the powders was determined against both Gram-positive (methicillin-sensitive (MS) and methicillin resistant (MR) *Staphylococcus aureus*) and Gram-negative (*Escherichia* coli and *Pseudomonas aeruginosa*) bacteria. The Cu-doped biomaterials exhibited a strong antibacterial activity against MSSA, MRSA and *E. coli*, releasing approximatively 2.5 ppm after 24 h, whereas 10 ppm were required to induce an antibacterial effect against *P. aeruginosa*. This study also demonstrated that the culture medium used during experiments can directly impact the antibacterial effect observed; only 4 ppm of Cu^2+^ were effective for killing all the bacteria in a 1:500 diluted TS medium, whereas 20 ppm were necessary to achieve the same result in a rich, non-diluted standard marrow cell culture medium.

## 1. Introduction

Bone substitutes are biomaterials whose aim is to replace the bone and rebuild a deficient bone stock in a completely safe way. They can also act as prosthesis coatings to improve the bond between tissue and material [1]. Ideally, they must be bioactive, osteoconductive, osteoinductive and bioresorbable [2]. In different cases, the use of bone substitutes is necessary to fill a damaged area: non-contiguous fractures with a bone defect, bone infection or disease requiring bone tissue removal. Autografts and allografts are conventional substitute techniques, which show interesting characteristics. However, they present limits in terms of both quantity and quality, respectively, leading to the development of synthetic bone substitutes [3]. Among these, Biphasic Calcium Phosphates (BCP) are promising candidates for bone repair surgery because their chemical and mineral composition is very similar to that of bone tissues [4]. BCPs are bioceramics composed of a mixture of hydroxyapatite (HAp, Ca_10_(PO_4_)_6_(OH)_2_) and beta-Tricalcium Phosphate (β-TCP, Ca_3_(PO_4_)_2_) [5]. An interesting characteristic of the biphasic composition is their solubility complementarity: HAp is poorly resorbable whereas β-TCP is highly degradable, allowing the resorption rate to be adapted to clinical needs [6]. Furthermore, apatitic materials have a flexible structure capable of accepting many ionic substitutions in their composition, enabling adaptable synthesis by doping with an element of biological interest [7]. In addition to these properties, BCPs are interesting materials because it is possible to adjust their composition and, therefore, to control the kinetic release of the doping element [6].

As well as biocompatibility, another essential factor to control is the risk of infection after the implantation of a bone substitute, especially because infections in bone sites are difficult to treat due to their deep localization in the tissue and poor vascularity. Infections can lead to non-osteointegration and necrosis, and the consequences for the patient and society can be devastating [8,9]. With the increase in bacterial resistance to antibiotics [10], research that is focused on the development of new strategies and the use of metallic ions as antibacterial elements is intensifying.

Copper (Cu) is an essential trace element involved in several biological processes, which is also known for its antibacterial properties [11,12]. Considering its strong antibacterial properties and limited cytotoxicity, the Cu^2+^ copper cation appears to be a promising doping candidate to improve the behavior of the bioceramics used in bone repair surgery [13]. Radovanovic et al. (2014) [14], synthetized Cu-doped BCP powders (HAp/α-TCP), and the results indicated significant antibacterial activity after 24 h of incubation for the four tested microorganisms, *MSSA*, *E. coli*, *P. aeruginosa* and *C. albicans,* compared to undoped HAp/α-TCP. Recently, Bhattacharjee et al. (2019) [15]. prepared Cu-doped hydroxyapatite. Results indicated a significant reduction in bacterial viability for *E. coli* and Methicillin-sensitive *Staphylococcus aureus* (MSSA). All the studies related to the biological behavior of copper-doped materials (antibacterial, angiogenic and osteogenic), in anticipation of orthopedic clinical applications, were presented in a recent review [16]. More than a quarter of the presented papers in this review were published over the past three years, highlighting the interest of the scientific community for the topic.

The purpose of this study was to investigate the cytotoxicity and antibacterial properties of a series of Cu-doped BCP powders. The influence of the amount of copper and the sintering temperature on biological responses were evaluated. Cytotoxicity assays were performed in vitro with human-Mesenchymal Stems Cells (h-MSCs). The antibacterial activity of the powders was investigated against four bacterial strains of clinical interest: MSSA, Methicillin-Resistant *Staphylococcus aureus* (MRSA), *Escherichia coli* and *Pseudomonas aeruginosa* (two Gram-positives, and two Gram-negatives). In this study, the bioceramics were tested in the form of uncompacted powder in order to favor the impact of the copper dopant. In addition, the role played by the composition of the culture medium on the antibacterial effects is also underlined in this study (which may explain discrepancies in the literature as noted in the recent review [16]). This made it possible to evaluate the cytotoxicity and the antibacterial effect in the same culture medium, and consequently, with equivalent copper release rates: an important experimental variable which is generally not taken into account. Finally, the results obtained from copper-doped powders are also compared with tests carried out on culture media containing Cu^2+^ cations in order to highlight the beneficial contribution of the bioceramic doping.

## 2. Materials and Methods

### 2.1. Sol-Gel Synthesis of Copper-Doped BCP Samples

Chemical syntheses were performed using the sol-gel route described and used by Gomes et al. (2018) [7]. Briefly, Ca(NO_3_)_2_·4H_2_O (Sigma-Aldrich, Saint-Quentin-Fallavier, France) and P_2_O_5_ (Sigma-Aldrich) were separately dissolved in ethanol under stirring, and then mixed and refluxed at 85 °C for 24 h. The solution obtained was maintained at 55 °C for 24 h and the resulting gel was heated at 80 °C for 10 h to obtain a white powder. To synthesize Cu-doped samples, the required amounts of Cu(NO_3_)_2_·3H_2_O (Sigma-Aldrich) were added. Finally, the powder was heat-treated for 15 h at 600 °C, 900 °C and 1200 °C, influencing the HAp/β-TCP ratio, and the location and accessibility of the doping element [7]. Four doping rates were synthesized, assuming the insertion of Cu^2+^ cations in the interstitial crystallographic sites of the HAp phase as described in our previous study [7]. The targeted nominal sample compositions were Ca_10_(PO_4_)_6_(OH)_2_ (corresponding to the undoped series), Ca_10_Cu_0_._05_(PO_4_)_6_(OH)_1_._90_O_0_._10_, Ca_10_Cu_0_._10_(PO_4_)_6_(OH)_1_._80_O_0_._20_ and Ca_10_Cu_0_._20_(PO_4_)_6_(OH)_1_._60_O_0_._40_. The materials were mainly composed of the HAp phase, mixed with a small amount of β-TCP. Color samples were dependent on the sintering temperature: light grey for the series annealed at 600 °C, dark grey for those at 900 °C and dark purple for samples treated at 1200 °C. In this study, samples are labeled “*X*Cu-*T*” with *X* = 00, 05, 10 and 20, corresponding to the four doping rates (undoped samples with *x* = 0, and *x* = 0.05, 0.10 and 0.20, respectively, for the stoichiometric amount of copper in the doped Ca_10_Cu*_x_*(PO_4_)_6_(OH)_2-2*x*_O_2*x*_ samples with *X* = 100*x*) and *T* indicating the sintering temperatures of 600 °C, 900 °C and 1200 °C (leading to a total of twelve different powders). Regarding the behavior in solution of these powders, we show that copper is easily accessible for materials treated at 600 °C and 900 °C, and that this is much less the case for powders treated at 1200 °C. Copper ions are located at the HAp crystal surface for the *X*Cu-600 samples (i.e., directly accessible), substituted for calcium in the β-TCP structure for the *X*Cu-900 samples (i.e., easily accessible due to the solubility of β-TCP), and inserted into the HAp structure for the *X*Cu-1200 samples (i.e., poorly accessible due to the non-solubility of HAp) [7].

Specific surface areas were obtained using MicroActive Software for a TriStar II PLUS Version 2.03 Instrument (Micromeritics, Merignac, France). Phase compositions were determined by X-ray powder diffraction (XRPD, X’Pert-PRO PANalytical, Malvern, UK) followed by Rietveld treatments to extract the respective amounts of HAp and β-TCP (this procedure was detailed in the related previous work [7], and the results will not be explained again here).

After synthesis, Cu-doped BCP powders were pre-treated by incubation in distilled water for 48 h at a concentration of 250 mg/L in order to remove CaO traces, which were responsible for deleterious pH increases as reported before [7]. Before biological evaluations, Cu-doped BCP samples were sterilized with dry heat in an oven at 180 °C for 2 h [17,18].

### 2.2. Cytotoxicity Evaluation

#### 2.2.1. Human Mesenchymal Stem Cells (h-MSCs): Isolation and Culture

The choice of h-MSVCs cells was made in order to be as close as possible to the clinical purpose of the material invested. H-MSCs were extracted from pieces of metaphysal cancellous bone, collected during hip arthroplasty on healthy patients who had signed an authorization for the use of their bone for research purposes. Bone pieces were collected in a solution of sterile phosphate-buffered saline (PBS) supplemented with 2% of heparin and transported directly to the culture lab. Samples were filtrated and washed with PBS. Bones were cut into small pieces and incubated for 15 min at 37 °C with 4 mL of minimum essential medium (MEM), 2 mL of PBS and 0.2 mL of collagenase (Stemcell). Samples were filtrated and then washed with PBS again. All the filtrates were mixed and centrifuged, and cell pellets were suspended in a standard marrow cell culture medium (MEM supplemented with gentamycin at 4 µg/mL, sodium pyruvate 1%, vitamins 1%, nonessential amino acids 1%, and fetal bovine serum 10%). Cells were plated in units of 20 × 10^6^ cells in 25 cm^2^ tissue culture flasks and incubated at 37 °C with 5% humidified CO_2_. After 3 days, the non-adherent cells were harvested and removed with two gentle rinses with PBS. Adherent h-MSCs were cultured with a weekly change of medium and expanded through one of three passages before being collected by trypsinization.

#### 2.2.2. Cytotoxicity Evaluation of Cu-Doped Powders with MTT Assay

H-MSCs were seeded in a 24-well plate (5 × 10^4^ cells/well) with 1.7 mg each of Cu-doped and undoped sterilized powders and 1 mL of standard marrow cell culture medium (the solid/liquid ratio was chosen to target a release rate of copper of the order of 10 ppm). Cells cultured without powders were used as a positive control. For negative control 30 µL of cycloheximide (Sigma-Aldrich, cycloheximide solution, 100 mg/mL in Dimethyl sulfoxide—DMSO, Sigma-Aldrich, Saint-Quentin-Fallavier, France) were added 24 h before the MTT (3-(4,5-dimethylthiazol-2-yl)-2,5-diphenyl tetrazolium bromide) assay. After 3, 7 and 15 days, the mitochondrial activity of h-MSCs was evaluated with an MTT assay. Concerning assessments of cytotoxicity at day 15, the cell culture medium was refreshed at day 7 so that the cells would be properly maintained and in order to avoid a potential cytotoxic effect, which would have come from the fact that the cell culture medium had not been renewed for 15 days.

For the MTT assay, 100 μL of the MTT reagent (Sigma-Aldrich) at a concentration of 5 mg/mL in PBS was added to each well, and the plates were then incubated at 37 °C with 5% of CO_2_. After 3 h, each well was carefully removed without damaging the cells at the bottom and 500 µL of DMSO was added to each well. The plates were incubated in the dark with gentle shaking for 40 min to ensure a complete lysis of the cells. Finally, the contents of the wells, except for the powder, were transferred to new wells, and the optical density (OD) was measured at 570 nm and 690 nm with a ’Spark 10M TECAN spectrophotometer (Tecan, Männedorf, Switzerland) and Magellan™ software (version 1.1, Tecan, Männedorf, Switzerland).

#### 2.2.3. Cytotoxicity Evaluation of Cu^2+^ Ions in Standard Marrow Cell Culture Medium with MTT Assay

A primary copper solution at 3 mg/mL in a standard marrow cell culture medium was prepared from Cu(NO_3_)_2_.3H_2_O (Sigma-Aldrich) and used to test the cytotoxicity of different concentration of Cu^2+^ ions. H-MSCs were seeded in 24-well plate (5 × 10^4^ cells/well) in standard marrow cell culture medium with copper concentration of 0 ppm, 5 ppm, 10 ppm, 15 ppm, 20 ppm and 25 ppm. Cells cultured without copper (0 ppm) were used as positive control. After 3, 7 and 15 days, an MTT assay was used to evaluated mitochondrial activity as previously described (Section 2.2.2.).

### 2.3. Antibacterial Properties

#### 2.3.1. Bacterial Strains and Growth Conditions

Four bacterial strains were used. Two Gram positive clinical strains, a MSSA strain isolated from a patient with osteoarticular infection after total knee replacement [19], and an MRSA isolated from an osteoarticular infection, were collected in a Lyon (France) hospital. Two Gram-negative bacilli were also tested: *E. coli* (ATCC^®^ 25922™) and *P. aeruginosa* (ATCC^®^ 27853™). The complementarity of the two origins of the tested strains makes it possible to combine aspects relating to practical situations encountered in clinical applications (strains isolated from patients) and ease of comparison with related studies from the literature (ATCC strains).

Precultures were obtained by overnight growth at 37 °C in 5 mL of TS medium (Tryptic Soy broth without dextrose). Bacterial concentrations were evaluated by measuring the optical density (OD) at 620 nm.

#### 2.3.2. Antibacterial Activity of Cu^2+^ ions in 1:500 Diluted TS Medium

A primary copper solution at 3 mg/mL in a 1:500 diluted TS medium (from the standard JIS Z 2801) was prepared from Cu(NO_3_)_2_·3H_2_O (Sigma-Aldrich) and used to test the different concentrations of Cu^2+^ ions. Antibacterial experiments were carried out in 24-well culture plates, starting with a bacterial inoculum of 10^3^ CFU/mL (Colony-Forming Unit/mL) in a 1:500 diluted TS medium. Copper concentrations ranging from 0 ppm to 5 ppm were tested with MSSA, MRSA, and *E. coli*, and from 0 ppm to 10 ppm with *P. aeruginosa*. Copper was added to each well and mixed with 1 mL of the bacterial suspension (10^3^ CFU/mL). Control conditions consisted of a bacterial suspension without any added copper. The number of viable bacteria in the inoculum was determined by plating 100 µL of the suspension on TS agar plates and determining the CFUs after 24 h of incubation at 37 °C. After 24h of incubation in the microtiter plates, 5 min of sonication was performed and repeated twice, and the number of remaining viable bacteria was determined by plating 100 µL of the suspension or serial dilutions of the suspension on TS agar plates, further incubated at 37 °C for 24 h. Up to 1.24 × 10^8^ CFU/mL were counted on the positive controls after 24 h of incubation (about 10^7^ CFU/mL on average), clearly indicating that the bacteria were in an exponential phase, leading to bacterial inoculates large enough to satisfy the statistical analysis.

#### 2.3.3. Antibacterial Activity of Cu-Doped BCP Powders in 1:500 Diluted TS Medium

Experiments were performed as described with Cu^2+^ copper ions solutions. A total of 6.8 mg of each powder were mixed with 1 mL of bacterial suspension at 10^3^ CFU/mL in each well of the 24-microtiter plate. The number of viable bacteria after 5 h and 24 h of incubation was measured by plating samples on TS agar plates as described previously. For these series of tests with BCP powders, up to 5.15 × 10^9^ CFU/mL were counted on the positive controls after 24 h of incubation (about 10^8^ CFU/mL on average).

#### 2.3.4. Antibacterial Activity against MSSA of Cu^2+^ Ions in Standard Marrow Cell Culture Medium (i.e., a Non-Diluted Medium)

In order to evaluate the influence of the culture medium used and the impact of its nutrient richness on the antibacterial properties of copper, a similar experiment as the one presented in Section 2.3.2. was performed but with a non-diluted standard marrow cell culture medium, that is to say, the supplemented medium used during cytotoxicity experiments (Section 2.2.1) without gentamycin, so as to allow bacterial growth. Copper concentrations ranging up to 20 ppm were tested against MSSA.

### 2.4. Measurement of Cu^2+^ Copper Ions Concentration

Ionic releases from the bioceramics during cytotoxicity and antibacterial evaluations were measured by Microwave Plasma–Atomic Emission Spectroscopy (4200 MP–AES from Agilent, Agilent, Les Ulis, France). Sampling was performed directly during the experiments, so the measurement conditions correspond exactly to those used during the tests. Calibration samples were prepared using a 1000 µg/mL Cu ion normadose (Agilent Technologies) diluted in the corresponding solvent (cell culture medium or bacterial culture medium). Samples were measured after dilution at a 1:10 ratio in HNO_3_ 2% solution. The two most intense emission wavelengths of Cu (324,754 nm and 327,395 nm) were selected to carry out the measurements.

### 2.5. Statistical Analysis

Results presented in this paper were obtained after 3 technical and 3 biological replicates (n = 9). The antibacterial effects of Cu-doped BCP powders were expressed in the form of a logarithmic difference between the bacterial concentrations obtained with the evaluated powders and the control without powder. The formula used was as follows: Logarithmic difference = log (powder tested) − log (control). For practical reference, a logarithmic difference of −3 log indicates a bacterial mortality of 99.9%, and must be considered effective for antibacterial application.

Statistical analyses were performed using the Mann−Whitney non-parametric test followed by the Bonferroni correction with *p* < 0.05 considered as being statistically significant.

## 3. Results

### 3.1. Synthesis and Characterization of Cu-Doped BCP Powders

The doping mechanism and chemical compositions of the powders used in this paper were previously described using the Rietveld method in the paper by Gomes et al. (2018) [7]. Some of the recorded X-ray powder patterns are shown in Figure S1 (Supplementary Information), and Rietveld analysis results are in agreement with our previous study. The extracted weight amounts of the two HAp and β-TCP phases are reported in Table 1, with the corresponding specific surface area for the three series of sintering temperatures (600 °C, 900 °C and 1200 °C). With increasing annealing temperature, the specific surface area of the powders decreased (as expected) and the HAp phase, which was already predominant, increased at the expense of the β-TCP phase, with almost only the HAp phase for the series at 1200 °C. The materials whose biological properties are evaluated in this paper are exactly the same as those of our previous paper, which detailed the material aspect in detail, and, in particular, the mechanism of copper incorporation according to the heat treatment [7]. This part will, therefore, not be detailed again and the reader is invited to refer to our previous paper [7] (which must be considered the 1st part of this study), our related article [20] and the recent paper of Bazin et al. (2021) [17]. In summary, the following conclusions should be kept in mind: materials treated at 600 °C and 900 °C easily release copper dopants, unlike materials treated at 1200 °C. This is explained by the different locations, at the atomic scale, of the copper atoms: adsorbed on the ceramic grains surface at 600 °C, substituting the Ca^2+^ cations of the β-TCP phase (of high solubility) at 900 °C, and inserted in the hexagonal channel in the HAp structure (of low solubility) at 1200°C. The values in Table 1 indicate that the physicochemical characteristics of powders are only dependent on the temperature and not on the amount of Cu doping: the values for the *X*Cu-600 series, then the *X*Cu-900 and *X*Cu-1200 series, are almost invariant for a given *X* value. Thus, the comparison of behaviors in a biological environment within a series will allow us to highlight the effect of the doping rate, while a comparison between the series will be significant for the localization of the dopant in the material (i.e., dopant release rate associated with copper accessibility).

On the other hand, particle size remains relatively homogenous between powders (see particle size distributions in Supplementary Information Figure S2): the size of particles was of the order of a few tens of micrometers (up to 100 µm for the *X*Cu-900 and *X*Cu-1200 series, and up to 300 µm for the *X*Cu-600 series, taking into account the non-agglomerated powders distributions obtained by ultrasonic separation) with the presence of fines (two families centered at 1 µm and 4 µm) due to the grinding effect, and a larger particles family (centered around 80 µm, 50 µm and 25 µm for the *X*Cu-600, *X*Cu-900 and *X*Cu-1200 series, respectively). An agglomeration effect is also evidenced by the difference of the granulometric distribution observed in the absence of ultrasonic dispersion: the family of large particles is then centered around 105 µm for the two *X*Cu-600, *X*Cu-900 series, and around 45 µm for the *X*Cu-1200 series. This particle size evolution linked to grinding is related to the sintering temperature, which make the ceramic more brittle, and must be uncorrelated from the specific surface evolution. The main difference between samples comes from the more brittle nature of the powders with the increase in the sintering temperature, resulting in a higher proportion of fines at 1200 °C. Since particle sizes of families of fines were equivalent between powders (only the fine/large particle ratio increased at 1200 °C), the physical particle/cell interactions should not differ between samples, and therefore should not lead to a bias in biological observations. SEM (Scanning Electron Microscopy) observations (Figures S3 and S4) made it possible to verify that all the samples presented identical particle morphologies: slightly angular due to manual grinding.

For information, a future paper will be dedicated to related ceramics, sintered in the form of a pellet, in order to highlight the effect of shaping on biological responses.

### 3.2. Cytotoxicity Measurements

#### 3.2.1. Cytotoxicity Measurements on Cu-doped BCP Powders

**Figure 1:**
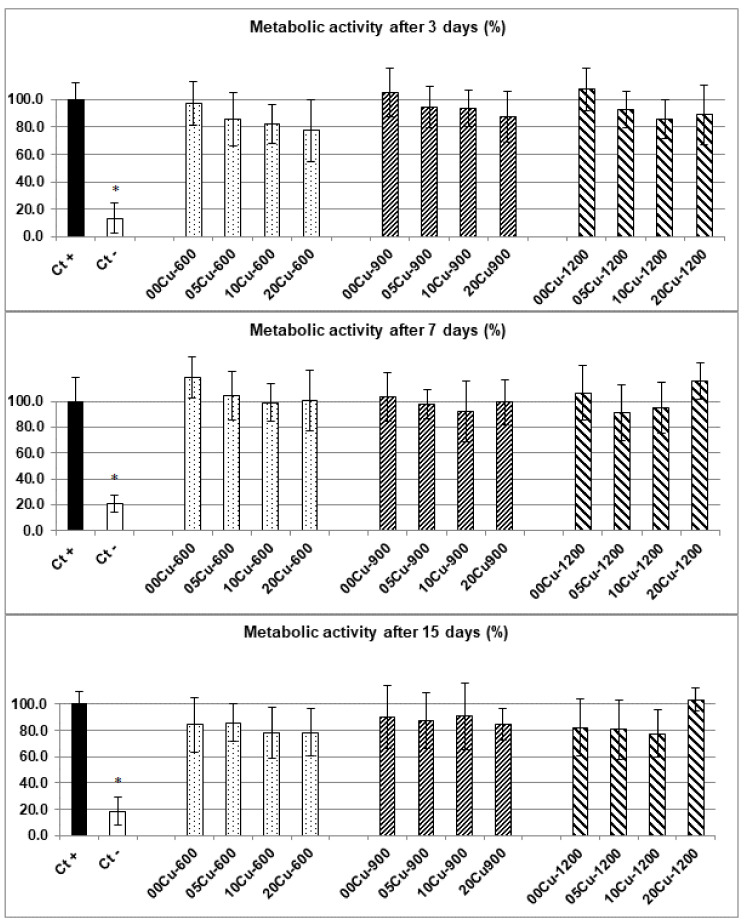
Metabolic activity (%) of h-MSCs after 3 (**top**), 7 (**middle**) and 15 (**bottom**) days of culture for the different Cu-doped BCP powders. Ct+ corresponds to the positive control and Ct- corresponds to the negative control. Error bars represent the standard mean error from n = 9 replicates. Results which are statistically different compared to the control (with *p* < 0.05) are indicated by “*”.

Results obtained from the MTT assay are presented in Figure 1 and the corresponding values are listed in Table S1. The positive control corresponds to 100% metabolic activity. At Day 3, the metabolic activities of all samples were over 80% except for sample 20Cu-600, with 77.6% (see Supplementary Information Table S1). However, the statistical analysis, based on nine replicates, indicated that there was no associated deleterious effect. At Day 7, all metabolic activity values were over 90%, and at Day 15, all results were over 80% except for 10Cu-600, 20Cu-600 and 10Cu-1200 with 77.9%, 78.4% and 77.8%, respectively. No significant difference with the positive control was shown, except for the negative control, which was significantly lower compared to all conditions.

The concentrations of Cu^2+^ released by the powders at Days 3, 7 and 15 are listed in Table 2. These concentrations were obtained directly by sampling the culture media during the cytotoxicity experiments. For measurements at Day 15, the cell culture medium had been refreshed at Day 7, so the indicated concentrations correspond to the release after the day of refreshment (i.e.*,* between day 7 and day 15) and the sum is indicated in brackets. The release rates shown in Table 2 highlight three main fact: (i) the release rate in the liquid is almost proportional to the solid doping rates in a *X*Cu-*T* series, (ii) the release rates are substantial for the two *X*Cu-600 and *X*Cu-900 series and low for the *X*Cu-1200 series (as predicted by the dopant atomic location from the material characterization [7]), and (iii) on the 3rd day, the equilibrium was reached because no significant evolution in the following terms are identifiable.

Thus, these results indicated the absence of cytotoxicity problems emanating from our samples, neither because of the contact with ceramic particles, nor because of the quantities of copper released.

#### 3.2.2. Cytotoxicity Measurements in Copper Containing Solutions

**Figure 2:**
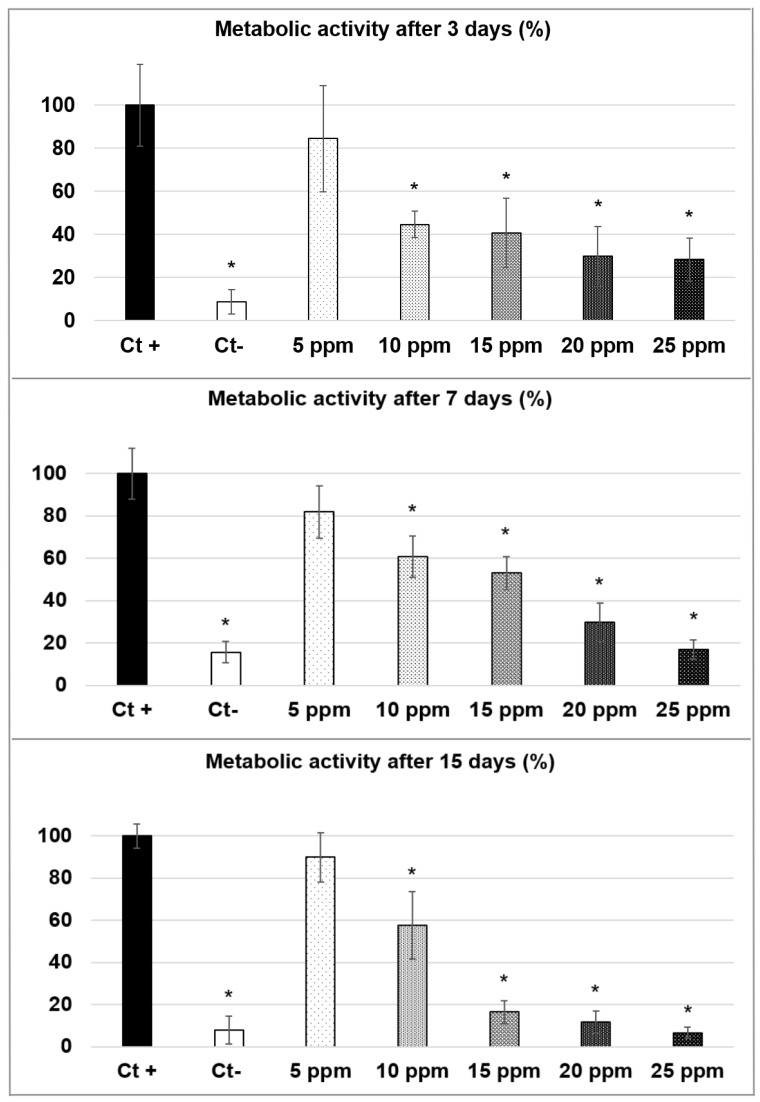
Metabolic activity (%) of h-MSCs after 3 (**top**), 7 (**middle**) and 15 (**bottom**) days of culture with copper containing standard marrow cell culture medium from 5 to 25 ppm. Ct+ corresponds to the positive control and Ct- corresponds to the negative control. Error bars represent the standard mean error from n = 9 replicates. Results which are statistically different compared to the control (with *p* < 0.05) are indicated by “*”.

In order to differentiate the investigated cytotoxicity of Cu-doped BCP powders from the intrinsic effect of the solvated Cu^2+^ cations alone, equivalent MTT assays were carried out on h-MSCs cultures in a standard marrow cell culture medium with Cu^2+^ adjuvant (from 5 ppm to 25 ppm of Cu^2+^—i.e., without the presence of BCP powders). The results are presented in Figure 2. Positive control (0 ppm) corresponds to 100% of metabolic activity. At Day 3, with 5 ppm of copper, no significant difference was observed in the metabolic activity of h-MSCs compared to the control without copper. On the other hand, there was a significant decrease in the metabolic activity of h-MSCs for upper concentrations with about 45% and 40% of metabolic activity obtained with 10 and 15 ppm, respectively, and 30% with 20 ppm and 25 ppm. The same results are observed at day 7, with no significant difference with 5 ppm of copper compared to the control and a significant decrease in the metabolic activity of h-MSCs for upper copper concentrations. Metabolic activities were 60%, 53%, 30% and 17% for 10, 15, 20 and 25 ppm respectively. At Day 15, results indicated no significant difference with 5 ppm and, again, significant decreases for upper copper concentrations. Metabolic activities were 58%, 16%, 12% and 7% for 10, 15, 20 and 25 ppm, respectively.

### 3.3. Antibacterial Activity of BCP Samples

#### 3.3.1. Antibacterial Activity of Cu-Doped BCP Powders against MSSA in 1:500 Diluted TS Medium

**Figure 3:**
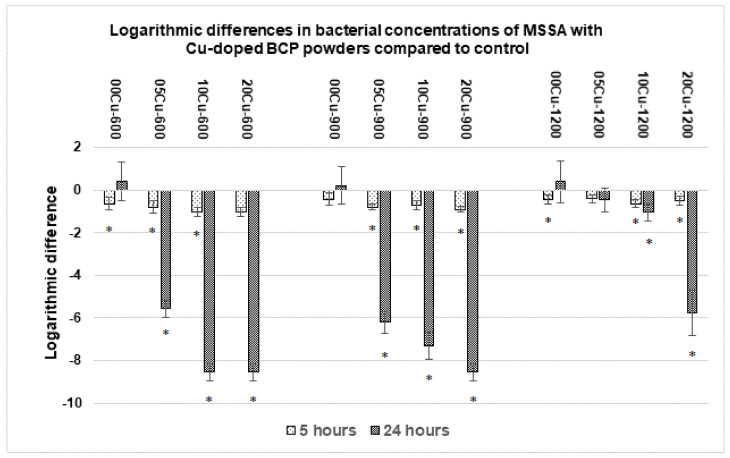
Concentrations of viable MSSA after incubation with Cu-doped BCP powders compared to the control at 5 h and 24 h. Results statistically different compared to the control (with *p* < 0.05) are indicated by “*”.

As indicated in Section 2.3.2, the use of the 1:500 diluted TS medium was justified by the standard JIS Z 2801 procedure dedicated for antibacterial products. The antibacterial assessment of Cu-doped powders was first performed for the MSSA strain and the results are presented in Figure 3. After 5 h of incubation, undoped and Cu-doped samples of the three series showed a decrease in bacterial concentration of about one log for both the *X*Cu-600 and *X*Cu-900 series and of about half a log for the xCu-1200 series. After 24 h of incubation, significant antibacterial activity was observed except for 05Cu-1200. The *X*Cu-1200 series behaved differently from the other two series. For the series sintered at 600 °C and 900 °C, a reduction in bacterial concentration of more than 5 log (corresponding to only 0.001 % of bacteria still alive) was observed in the presence of copper. The decrease even exceeded 8 log compared to the control for samples 10Cu-600, 20Cu-600 and 20Cu-900, reaching the detection threshold and corresponding to the death of all bacteria. The series sintered at 1200 °C showed antibacterial properties but which were less effective: samples 10Cu-1200 and 20Cu-1200 induced a significant decrease in bacterial concentrations, with a logarithmic reduction of −1.0 and −5.8, respectively. After 24 h of incubation, the three undoped 00Cu-*T* samples did not induce any reduction in the number of CFUs. For the *x*Cu-600 and *x*Cu-900 series, significant antibacterial effects were observed, even for the less doped 05Cu-600 and 05Cu-900 samples, and the effect increased with dopant concentration. Although the antibacterial effects were inferior for the series xCu-1200, the effect also increased with dopant concentration.

#### 3.3.2. Antibacterial Activity of Cu-Doped BCP Powders against the Three other Strains in 1:500 Diluted TS Medium

**Figure 4:**
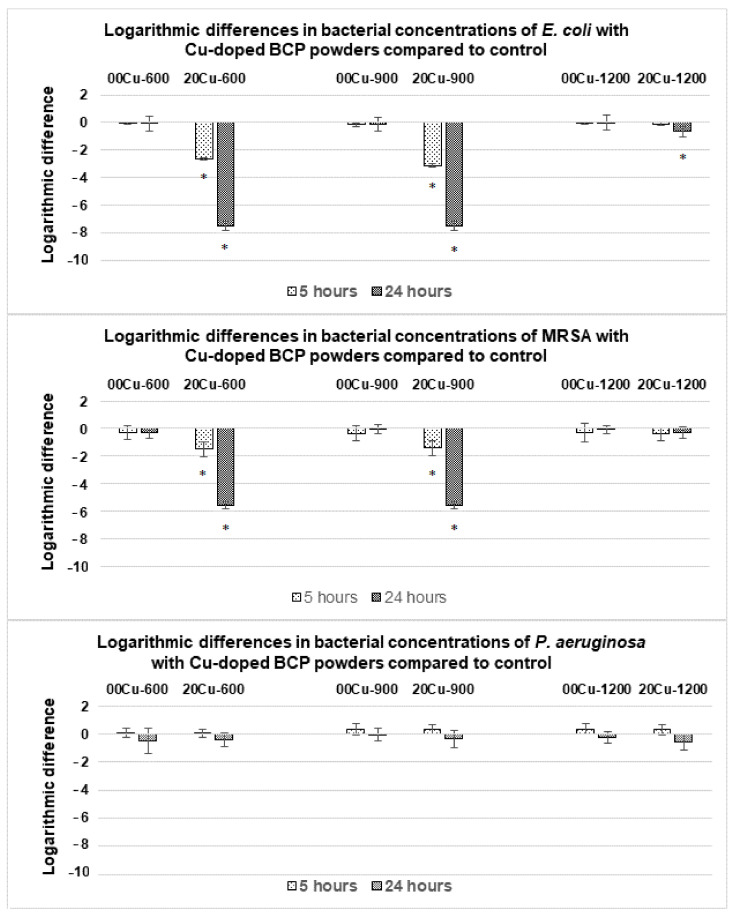
Logarithmic differences in bacterial concentrations of *E. coli* (**top**)*,* Methicillin-Resistant *S. aureus* (MRSA) (**middle**) and *P. aeruginosa* (**bottom**) with Cu-doped BCP powders compared to the control (undoped powders) after 5 h and 24 h. Results statistically different compared to the control (with *p* < 0.05) are indicated by “*”.

Assays were performed on the three other bacterial strains (MRSA, *E. coli* and *P. aeruginosa*) with 00Cu-*T* undoped powders and 20Cu-*T* doped powders. The results of the previous Section 3.3.1 have shown that the use of these two powders was significant to highlight an effect linked to Cu-doping. Results showed an antibacterial activity for 20Cu-600 and 20Cu-900 powders against *E. coli* within 5 h of incubation, and the number of viable bacteria was reduced by approximatively 8 log after 24 h (Figure 4, top), corresponding to the death of all the bacteria. Concerning the series sintered at 1200 °C, only a small antibacterial effect was observed after 24 h with the 20Cu-1200 powder (Figure 4, top). The antibacterial activity against the clinical strain of MRSA, after 5 h of incubation showed a reduction of ~1.5 log with 20Cu-600 and 20Cu-900, and after 24 h, no viable bacteria were detected with these two powders. The series sintered at 1200 °C did not present an antibacterial effect for MRSA (Figure 4, middle). No significant antibacterial activity of the Cu-doped powders was observed with *P. aeruginosa,* even after 24 h and regardless of the sintering temperature (Figure 4, bottom).

Sampling was performed during antibacterial assays to measure the concentration of copper released in the culture medium, and the results are listed in Table 3. Regardless of the strain tested, no variation in the copper release rate was observed. Powders containing higher dopant amounts released higher Cu^2+^ ion concentrations. Medium copper concentrations of the series treated at 600 °C were ~2.4 ppm after 5 h, and ~2.6 ppm after 24 h of incubation. For series treated at 900 °C, medium copper concentrations were slightly lower with ~1.7 ppm after 5 h and ~2.4 ppm after 24 h, showing a slower copper release kinetic but resulting in a similar solvated Cu^2+^ concentration after one day. Powders sintered at 1200 °C released lower amounts of copper with ~0.9 ppm and ~1.3 ppm after 5 h and 24 h of incubation, respectively.

#### 3.3.3. Antibacterial Activity of Copper Containing 1:500 Diluted TS Medium

**Figure 5:**
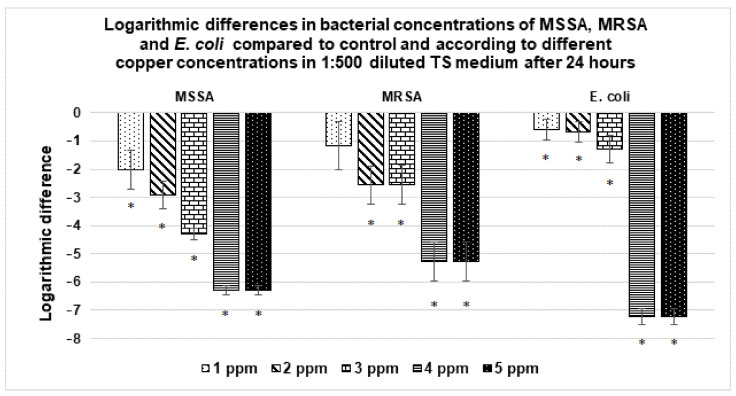
Variation in the bacterial concentration of MSSA, MRSA and *E. coli* with respect to different copper concentrations in 1:500 diluted TS medium after 24 h. Results statistically different compared to the control (with *p* < 0.05) are indicated by “*”.

**Figure 6:**
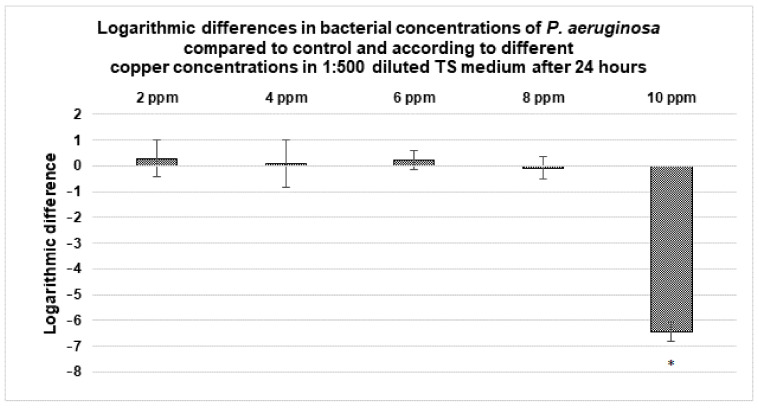
Variation in the bacterial concentration of *P. aeruginosa* with respect to different copper concentrations in 1:500 diluted TS medium after 24 h. Results statistically different compared to the control (with *p* < 0.05) are indicated by “*”.

Antibacterial assays were performed with a copper-containing 1:500 diluted TS medium (from 1 ppm to 10 ppm of solvated Cu^2+^, in the absence of BCP powders) over 24 h in order to investigate the antibacterial role intrinsic to Cu^2+^ cations without involving the BCP powder doping. The results are presented in Figure 5 (for the MSSA, MRSA and *E. coli* strains) and Figure 6 (for the *P. aeruginosa* strain). Copper ions had an antibacterial activity against MSSA, MRSA and *E. coli* already at 1 ppm concentration (Figure 5). These antibacterial activities increased with copper concentration and above 4 ppm, no viable bacteria were detected. Concerning *P. aeruginosa*, no effect was observed with 2, 4, 6 and 8 ppm. A total of 10 ppm was required to induce a reduction in the *P. aeruginosa* bacterial concentration (Figure 6).

#### 3.3.4. Antibacterial Activity against MSSA of Copper Containing Non-Diluted Media (Standard Marrow Cell Culture Medium)

**Figure 7:**
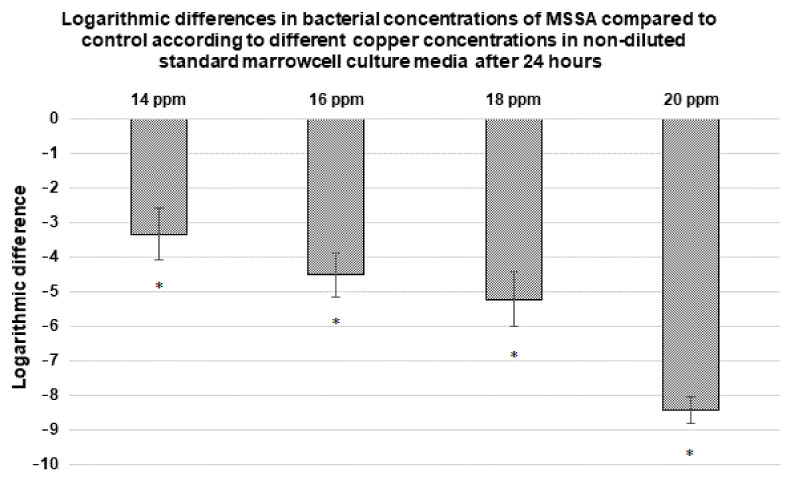
Variation in the bacterial concentration of MSSA with respect to different copper concentrations in a non-diluted standard marrow cell culture medium after 24 h. Copper concentrations below 14 ppm showed results similar to the control (0 ppm); therefore, bacterial concentrations values are not presented here. Results statistically different compared to the control (with *p* < 0.05) are indicated by “*”.

The antibacterial properties of copper (up to 20 ppm) were also assessed in a non-diluted standard marrow cell culture medium with the MSSA strain, and results are presented in Figure 7 (copper concentrations below 14 ppm showed results similar to the control; therefore, bacterial concentration values are not presented here). Concentrations of 14, 16 and 18 ppm of Cu^2+^ exhibited antibacterial activity with −3, −4.5 and −5 log reductions, respectively. With 20 ppm of copper, no viable bacteria were detected. The previous Section 3.3.3 indicated that a concentration of 4 ppm was enough to kill the same strain in the 1:500 diluted TS medium.

## 4. Discussion

Cu-doped BCP powders are biomaterials capable of releasing Cu^2+^ ions into their environment, inducing biological interactions. This study enabled us to observe the impact of the HAp/β-TCP ratio on the solubility of the material and so, on the dopant release rate. In our experiments, samples from the 600 °C series containing about 12% of the soluble β-TCP phase and samples from the 900 °C series with about 6% of the β-TCP phase released the highest amount of copper during biological experiments (Table 1, Table 2 and Table 3). Series sintered at 1200 °C, containing about 98% of the poorly soluble HAp phase, presented the lowest copper release rate. Even more important than the HAp/β-TCP ratio, it is necessary to take into consideration the dopant location at the atomic level in order to predict its rate of release. Our previous study [7] highlighted that thermal treatment at 600 °C leads to a location at the crystal surface; at 900 °C, copper substitutes for calcium into the more soluble β-TCP phase; and at 1200 °C, copper inserts the less soluble HAp structure. Thus, the dopant is easily accessible when sintering at 600 °C and 900 °C, and less accessible at 1200 °C, in accordance with values from Table 2 and Table 3. In addition, results clearly indicate that the amount of copper incorporated in the material during synthesis impacts the level of copper release for the three, 600 °C, 900 °C and 1200 °C, series (Table 2 and Table 3).

### 4.1. Cytotoxicity Evaluation

In this study, the cytotoxicity of Cu-doped BCP powders was evaluated with human Mesenchymal Stem Cells (h-MSCs), and none of the different Cu-doped BCP powders tested presented toxicity (Figure 1). Murine/rat cell lines and osteoblastic cells are often used in cytotoxicity assays of bone biomaterials [21,22,23,24] and the Cu^2+^ cytotoxicity usually presents dose-dependent responses according to cell types [25]. H-MSCs are more representative of the cell type directly in contact with biomaterials during the implantation procedure, so their use represents a suitable model for the development of functional synthetic implants.

Results from Cu^2+^ containing a standard marrow cell culture medium deviate from the observations extracted from the cultures with the Cu-doped powders. During these tests, only the concentration of 5 ppm of Cu^2+^ was found to have no cytotoxic effect on the h-MSCs cells (Figure 2). Indeed, 10 ppm of Cu^2+^ in solution induced a consequent loss of metabolic activity from the 3rd day (that continued at 7 and 15 days), whereas a quantity of 12 ppm released by the 20Cu-600 powder did not show cytotoxicity. Figure 2 also shows that the cytotoxic effect of Cu^2+^ introduced in a standard marrow cell culture medium is concentration dependent with a progressive decrease in the h-MSCs metabolic activity from 5 to 25 ppm of Cu^2+^. Concerning solutions with 15 ppm, 20 ppm and 25 ppm copper at 15 days, the metabolic activities were very low and comparable to the negative control corresponding to dead cells.

These two series of cytotoxicity evaluation highlighted the interest of the copper doping of the biomaterial, which induced improved biocompatibility of the Cu element. The presence of the BCP materials make it possible to reduce the potential toxicity of copper. The BCP positive effect could be due to the gradual release (due to kinetic) of copper by the powders compared to a copper concentration directly applied upstream of the culture with h-MSCs. The positive impact of BCP could also be explained by the associated and simultaneous release of calcium and phosphate (joint effect) by the material. To our knowledge, the study of the cytotoxicity of a range of copper concentrations with h-MSCs has never been described. Results observed in this study demonstrate the complexity of studying the biological properties of biomaterials and support the importance of measuring the amount of dopant released in biological media during experiments. Our measurements showed that a release rate of 12 ppm of copper by a Cu-doped BCP powder does not cause toxicity toward human MSCs.

### 4.2. Antibacterial Evaluation

Antibacterial assays demonstrated that the Cu-doped BCP powders used in this study had a strong antibacterial effect against clinical bacterial strains, including MRSA and MSSA isolated from osteoarticular infections. The use of clinical isolates is likely to provide a better overview of situations encountered in current medical practice [26,27]. Knowing that the increase in bacterial resistance to antibiotics is a major health issue [10], these results represent a promising solution to counter this issue. Regarding the tested bacterial strains, American Type Culture Collection (ATCC) strains (*E. coli and P. aeruginosa*) are commonly used, and this enables results to be easily compared between studies. The various tests carried out in our studies made it possible to highlight the complexity of the problem involved, as described in the subsections below.

#### 4.2.1. Antibacterial Activity of Cu-Doped BCP Powders in 1:500 Diluted TS Medium

The first series of tests made it possible to show the antibacterial effectiveness of the copper-doped bioceramic against the MSSA strain (Figure 3). The measurements at 24 h indicate the complete death of MSSA, provided that the copper release is sufficient. Indeed, BCP powders sintered at 1200 °C, which only release very little copper in the medium (Table 3), showed a much less marked efficiency, contrary to the two other series with copper release rates approaching 3 ppm. Results from the twelve powders tested against MSSA highlighted that the antibacterial effect is copper-doping-rate dependent. The 20Cu-*T* powders were more effective than 10Cu-*T*, and then 05Cu-*T* (even though the undoped 00Cu-*T* powders have not shown efficacy). Figure 3, combined with values from Table 3, allows to complement with the concept of the action-time necessary for the elimination of the strain of bacteria. After 5 h of culture with the 20Cu-600 powder, even though the release of copper reached 2.7 ppm, the logarithmic difference was not, or barely, significant.

The following series of tests, on the other three bacterial strains, made it possible to confirm these results while pointing out the sensitivity of the strains (Figure 4). The antibacterial property of the Cu-doping was maintained on MRSA and *E. coli*. On the other hand, no significant reduction in bacterial viability was observed on *P. aeruginosa*. This opportunistic pathogen is known to be highly resistant, being able to survive drastic osmolarity changes and to develop multiple mechanisms of antimicrobial resistance [28,29]. These discrepancies highlight the importance of measuring the amount of copper released during experiments to determine the potentially dose-dependent effects for each bacterial strain tested.

#### 4.2.2. Antibacterial Activity of Cu^2+^ Ions in 1:500 Diluted TS Medium

In order to highlight the dose-dependent notion, and to overcome—again—the BCP material effect, new series of tests were carried out on solutions (the same 1:500 diluted TS medium) with low Cu^2+^ content (up to 10 ppm). Again, *P. aeruginosa* expressed more resistant behavior than the three other strains. A total of 10 ppm of Cu^2+^ were necessary to kill *P. aeruginosa* (Figure 6), whereas less than 4 ppm were effective against MSSA, MRSA and *E. coli* (Figure 5).

The material effect was also slightly present here. While less than 3 ppm of copper released was sufficient to kill the three strains (Table 3, Figure 3 and Figure 4), it was necessary to reach 4 ppm in the absence of BCP powder (for the same 24 h deadline). The difference being little marked here, it appears that the antibacterial effect is indeed intrinsically linked to Cu^2+^ cations (released in the medium, and perhaps also by contact with the Cu-doped powder surface).

#### 4.2.3. Antibacterial Activity against MSSA of Copper Containing Non-Diluted Standard Marrow Cell Culture Medium

A last series of tests allowed to probe the importance of the biological environment in which experiments are performed. The antibacterial activity against MSSA was tested in the standard marrow cell culture medium used for the cytotoxicity evaluation. In the diluted (and consequently poor) 1:500 TS medium, 4 ppm of Cu^2+^ were effective to kill all MSSA bacteria, whereas in a rich standard marrow cell culture medium, 20 ppm were necessary to reach the same effect (Figure 5 and Figure 7). The antibacterial properties of copper probably depend on a balance between the amount of copper required for the lysis of bacteria and the division rate of the bacteria, directly related to the richness in nutrients of the environment. From material point of view, the microstructure will also impact exchanges with the biological environment [17]. To our knowledge, the importance of considering the culture medium used has never been described in the literature; however, it should be taken into account when making comparisons between different studies from the literature as mentioned in the recent review [16].

Copper, besides being an essential trace element required for human body health, is also involved in numerous biological functions and several metabolic processes, such as angiogenesis and osteogenesis, two essential and related processes of wound-healing after bone-substitute implantation [11,30,31]. Barralet et al. (2009) [32] synthesized a copper-adsorbed macroporous scaffold and showed that low amounts of copper promote micro-vessel formation and the wound-healing process in mice Kong et al. (2014) [33] demonstrated that Cu ions contained in a calcium silicate bioceramic cause an increase in the vascularization of Human Umbilical Vein Endothelial Cells (HUVEC) and Human Dermal Fibroblasts (HDF) in co-culture and VEGF expression, attesting to a pro-angiogenic effect. Ewald et al. (2012) [34] observed an enhancement of the expression of bone-specific proteins in osteoblastic cells seeded on a scaffold loaded with Cu ions. Recently, Zhang et al. (2020) [35] synthesized a Cu-substituted dicalcium silicate cement and showed that the quantitative new bone formation was significantly higher with Cu cement than for undoped cement. Thus, the use of materials doped with copper ions, able to both prevent infections and promote wound healing, represents a promising research subject. In spite of these positive aspects, due to the contribution of copper for integration of the biomaterial (antibacterial, angiogenesis and osteogenesis necessary for wound-healing), it is necessary to proceed to test in real conditions (on animals) in order to fully understand the behavior in the real biological environment of such Cu-doped implants.

## 5. Conclusions

The aims of this work were to investigate the biological properties of Cu-doped BCP powders and the influence of their chemical characteristics on the results observed. For these purposes, the present study evaluated the cytotoxicity and the antibacterial properties, both combined to the release rate of Cu^2+^ ions, of a set of twelve Cu-doped BCP powders.

Concerning the chemical characteristics of the powders, this study allowed us to describe the impact of the sintering temperature on the ratio of HAp and β-TCP phases and on the atomic location of the copper, both influencing the accessibility of the doping element during contact with a biological medium. The proportion of the poorly soluble HAp phase increases with the annealing temperature, leading to a lower copper release rate. Furthermore, the amount of copper incorporated in the material during synthesis impacts the level of copper release. These features represent a major factor, which can easily be controlled during synthesis in order to adapt the material to the biological needs. Cytotoxicity evaluation clearly demonstrated that all the Cu-doped BCP powders exhibited no toxicity against h-MSCs, which is an essential step in the implantation of a bone substitute. Copper ions released by the powders exhibit a strong antibacterial activity against two clinical strains of *S. aureus*, including one resistant to methicillin, and against *E. coli*. Results indicated that a higher Cu^2+^ concentration was required to show an antibacterial effect against *P. aeruginosa*. Complementary results indicated that the antibacterial properties of copper depend on a balance between the copper concentration and the composition of the biological fluids, the latter impacting the copper release rate and the bacterial strains survival resistance.

In conclusion, this work enabled the development of promising biomaterials, which are biocompatible with strong antibacterial properties and adjustable synthesis, in order to meet biological needs. A study of complementary biological properties (e.g., angiogenesis and osteogenesis) would further enable the bio-adaptation of bone substitutes and contribute to the development of bone tissue engineering.

## Figures and Tables

**Table 1:** Specific area and mineral composition (weight percent, wt %) of the three series of sintering temperatures (*X* = 00, 05, 10 and 20).

Sample Series	Specific Surface Area (m^2^/g)	Composition (wt %) *	
		HAp	β-TCP
***X*Cu-600**	**~14.5**	**~88**	**~12**
00Cu-600	14.8	86	14
05Cu-600	16.7	88	12
10Cu-600	12.4	90	10
20Cu-600	13.7	90	10
***X*Cu-900**	**~3.5**	**~94**	**~6**
00Cu-900	3.3	96	4
05Cu-900	3.5	93	7
10Cu-900	4.1	94	6
20Cu-900	3	93	7
***X*Cu-1200**	**~1.0**	**~98**	**~2**
00Cu-1200	1.2	99	1
05Cu-1200	0.5	98	2
10Cu-1200	1	98	2
20Cu-1200	0.6	97	3

* values rounded to the unit, without considering the presence of lime (which never exceeded 1.5 wt %).

**Table 2:** Average values (from the nine replicates) of Cu^2+^ release (ppm) in standard marrow cell culture medium measured by MP-AES during the cytotoxicity evaluation of Cu-doped bioceramics.

Samples	Cu^2+^ Concentration (ppm)		
	Day 3	Day 7	Day 15 *
05Cu-600	2.4	2.8	0.9 (3.7)
10Cu-600	5.1	4.7	2.3 (7.0)
20Cu-600	11.5	12.0	3.5 (15.5)
05Cu-900	2.5	2.4	0.6 (3.0)
10Cu-900	5.2	5.5	1.2 (6.7)
20Cu-900	10.0	9.4	2.4 (11.8)
05Cu-1200	0.5	0.7	0.4 (1.1)
10Cu-1200	0.7	0.8	0.4 (1.2)
20Cu-1200	1.5	1.7	0.6 (2.3)

* For measurements at Day 15, the cell culture medium had been refreshed at Day 7, so the concentration indicated corresponds to the release between Day 7 and Day 15 (the sum with the Day 7 measurement is indicated in brackets).

**Table 3:** Average values (from the nine replicates realized on the four strains) of Cu^2+^ release (ppm) in 1:500 diluted TS medium measured by MP-AES during antibacterial assays of the Cu-doped bioceramics.

Samples	Cu^2+^ Concentration (ppm)	
	5 h	24 h
05Cu-600	2.2	2.2
10Cu-600	2.4	2.7
20Cu-600	2.7	2.8
05Cu-900	1.7	2.1
10Cu-900	1.5	2.2
20Cu-900	1.9	2.8
05Cu-1200	0.7	1.0
10Cu-1200	0.9	1.3
20Cu-1200	1.2	1.7

## Data Availability

The data presented in this study are available on request from the corresponding author.

## Supplementary Materials

The following are available online at https://www.mdpi.com/article/10.3390/ma14092393/s1, Figure S1. Examples of part (20° < 2θ < 60°) of the X-ray powder patterns recorded for the two 00Cu-T (top) and 10Cu-T series (bottom). Stars locate the main diffraction peaks of β-TCP. Figure S2. Particle size distributions for the undoped 00Cu-T series obtained without (a) and with (b) ultrasonic particles separation. Granulometric distributions were obtained with a Mal-vern Mastersizer 3000 with a Hydro EV automated wet dispersion unit. Figure S3a. SEM photos on the 00Cu-T undoped series with magnification from ×100 to ×2500. Bioceramics powders were deposited on a conductive carbon adhesive coating bonded on a sample holder. Samples were coated with a thin layer of gold using a DESK V (Denton Vacuum) metallizer. SEM observations were obtained using a Hirox SH-4000 M device operating at 20 kV. Figure S3b. SEM photos for the whole XCu-T samples with magnification ×800. Bioceramics powders were deposited on a conductive carbon adhesive coating bonded on a sample holder. Samples were coated with a thin layer of gold using a DESK V (Denton Vacuum) metallizer. SEM observations were obtained using a Hirox SH-4000 M device operating at 20 kV. Table S1. Values of metabolic activity (%) of h-MSCs obtained after 3, 7 and 15 days of culture with different Cu-doped BCP powders. Ct+ correspond to the positive control and Ct- corre-spond to the negative control. Data are given as mean ± standard error from n = 9 replicates.
