# Supplementary material for: Copper-Doped Biphasic Calcium Phosphate Powders: Dopant Release, Cytotoxicity and Antibacterial Properties
Source: Materials (Basel). 2021 May 4;14(9):2393. doi: 10.3390/ma14092393 (PMC8124198; doi:10.3390/ma14092393)
Supplement: Supplementary file 1 [file materials-14-02393-s001.zip › materials-1193556-supporting -for publish-update.pdf]

materials

Supplementary Materials

Copper-Doped Biphasic Calcium Phosphate Powders: Dopant Release, Cytotoxicity and Antibacterial Properties

Aurélie Jacobs [1], Guillaume Renaudin [1,*], Nicolas Charbonnel [2], Jean-Marie Nedelec [1], Christiane Forestier [2] and Stéphane Descamps [3]

1 Université Clermont Auvergne, Clermont Auvergne INP, CNRS, ICCF, F-63000 Clermont-Ferrand, France; aurel.jacobs@gmail.com (A.J.); jean-marie.nedelec@sigma-clermont.fr (J.-M.N.)
2 Laboratoire Microorganismes: Genome et Environnement, Université Clermont Auvergne, CNRS, F-63000 Clermont-Ferrand, France; nicolas.charbonnel@uca.fr (N.C.); christiane.forestier@uca.fr (C.F.)
3 Université Clermont Auvergne, Clermont Auvergne INP, CNRS, CHU Clermont, ICCF, F-63000 Clermont-Ferrand, France; s_descamps@chu-clermontferrand.fr
* Correspondence: guillaume.renaudin@sigma-clermont.fr

Citation: Jacobs, A.; Renaudin, G.; Charbonnel, N.; Nedelec, J.-M.; Forestier, C.; Descamps, S. Copper-Doped Biphasic Calcium Phosphate Powders: Dopant Release, Cytotoxicity and Antibacterial Properties. Materials 2021, 14, 2393. https://doi.org/10.3390/ma14092393

Academic Editor: Rene Buchet; Aivaras Kareiva

Received: 6 April 2021
Accepted: 2 May 2021
Published: 4 May 2021

Publisher's Note: MDPI stays neutral with regard to jurisdictional claims in published maps and institutional affiliations.

Copyright: © 2021 by the authors. Licensee MDPI, Basel, Switzerland. This article is an open access article distributed under the terms and conditions of the Creative Commons Attribution (CC BY) license (http://creativecommons.org/licenses/by/4.0/).

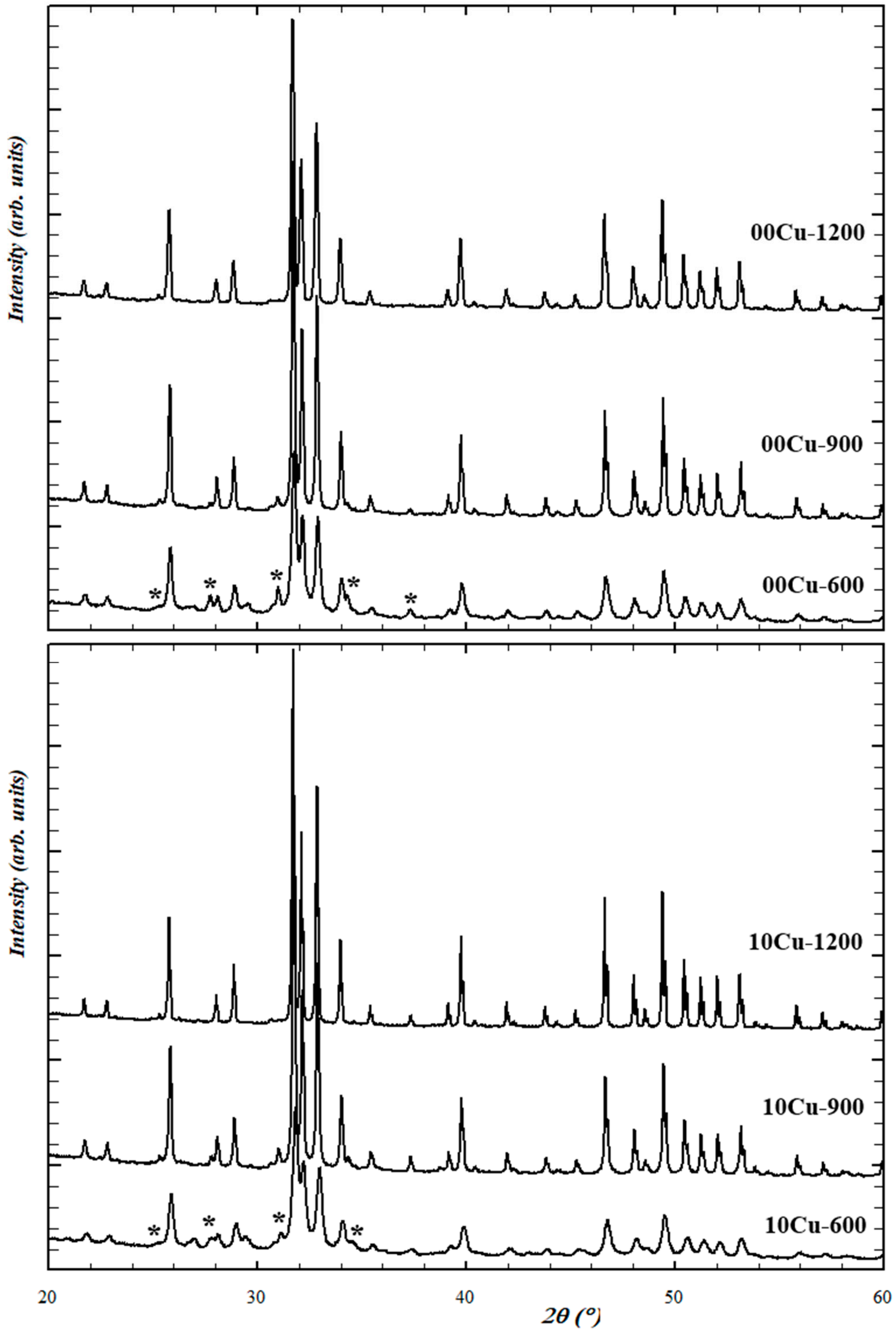

Figure S1. Examples of part (20° < 2θ < 60°) of the X-ray powder patterns recorded for the two 00Cu-*T* (top) and 10Cu-*T* series (bottom). Stars locate the main diffraction peaks of β-TCP.

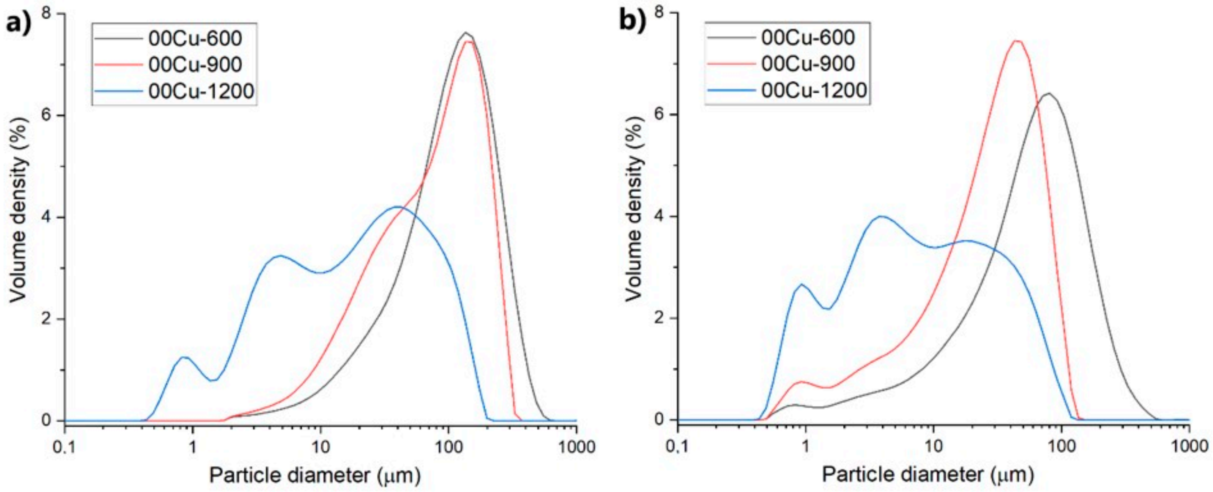

Figure S2. Particle size distributions for the undoped 00Cu-*T* series obtained without (**a**) and with (**b**) ultrasonic particles separation. Granulometric distributions were obtained with a Malvern Mastersizer 3000 with a Hydro EV automated wet dispersion unit.

**Magnif.**	**00Cu-600**	**00Cu-900**	**00Cu-1200**
100×	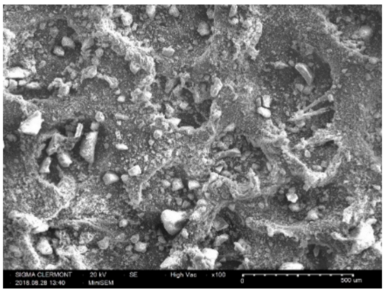	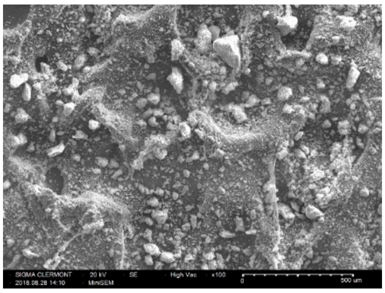	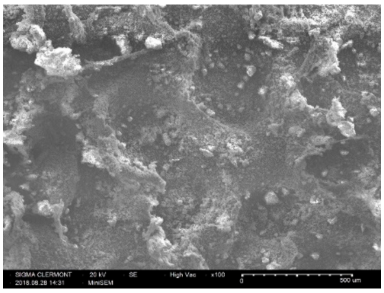
250×	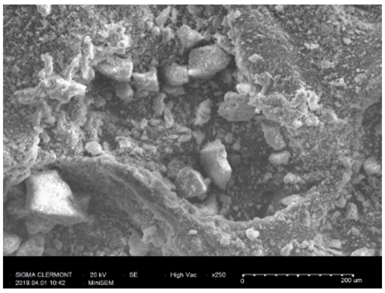	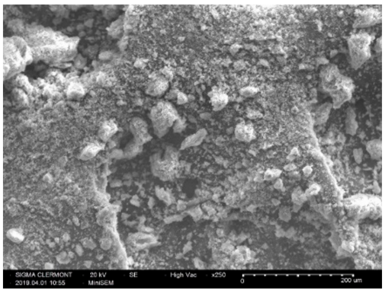	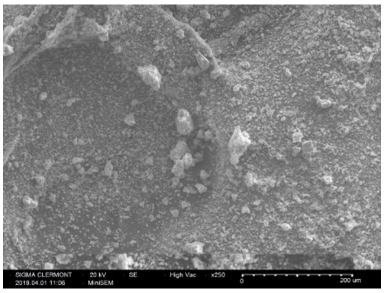
500×	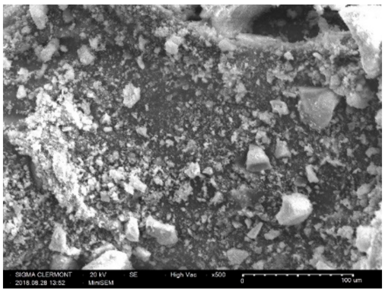	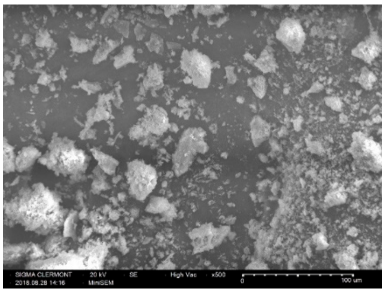	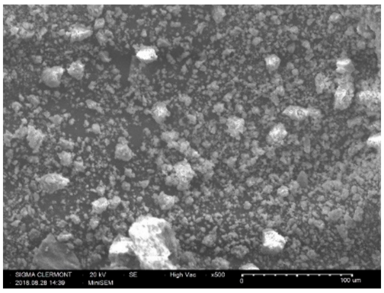

800×	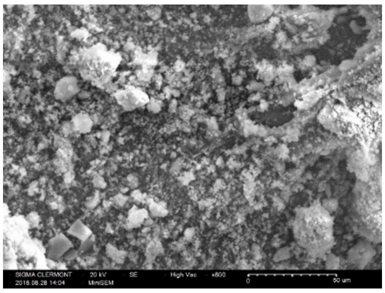	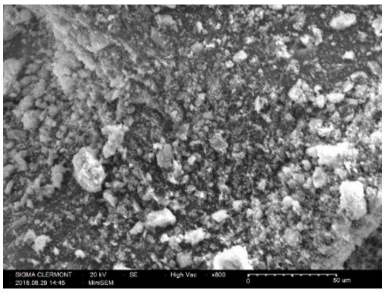	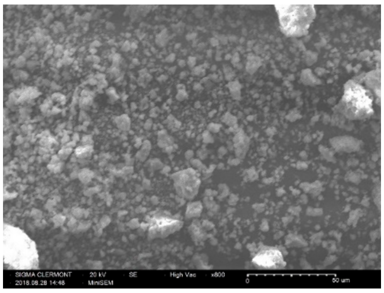
2500×	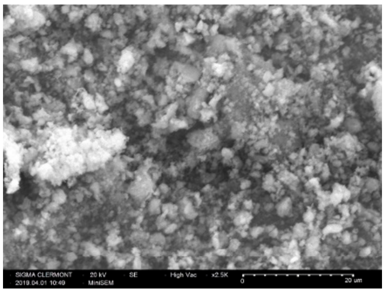	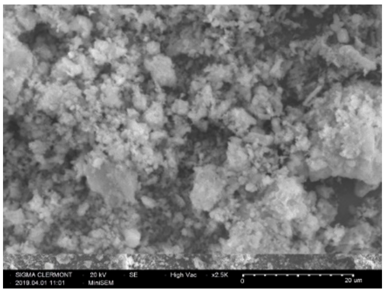	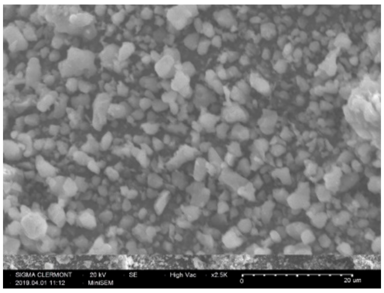

Figure S3. SEM photos on the 00Cu-*T* undoped series with magnification from 100× to 2500×. Bioceramics powders were deposited on a conductive carbon adhesive coating bonded on a sample holder. Samples were coated with a thin layer of gold using a DESK V (Denton Vacuum) metallizer. SEM observations were obtained using a Hirox SH-4000 M device operating at 20 kV.

**Sample**	***X*Cu-600**	***X*Cu-900**	***X*Cu-1200**
X = 00	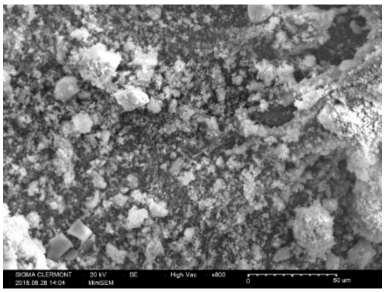	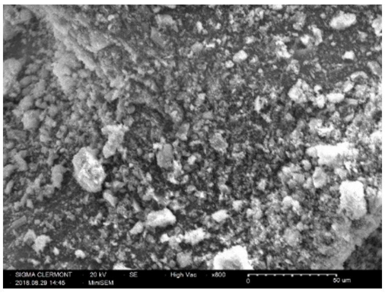	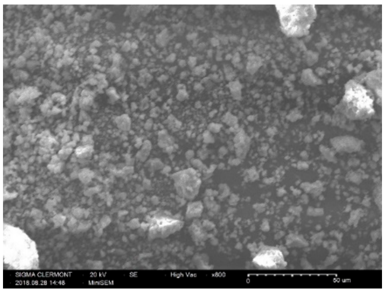
X = 05	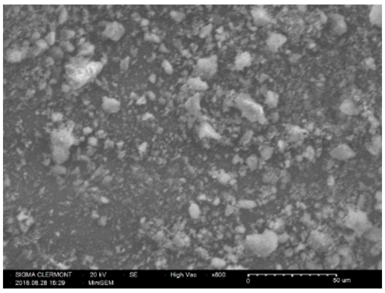	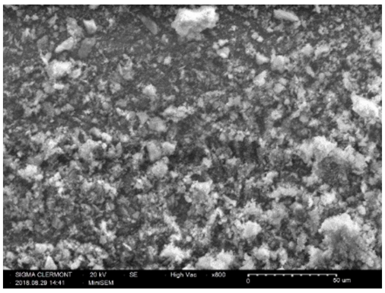	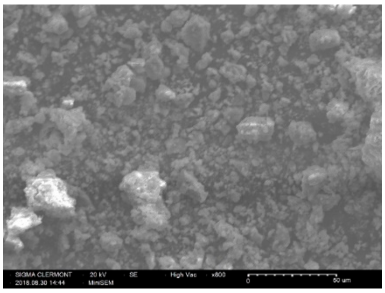
X = 10	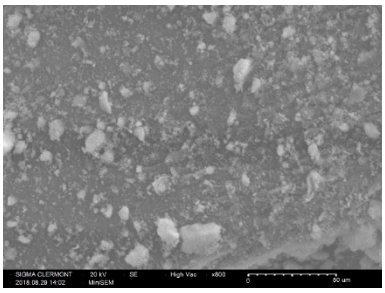	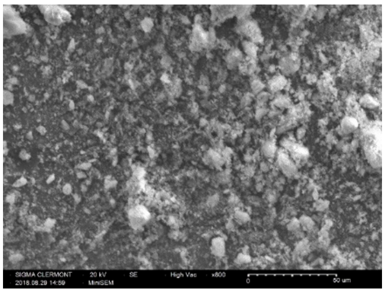	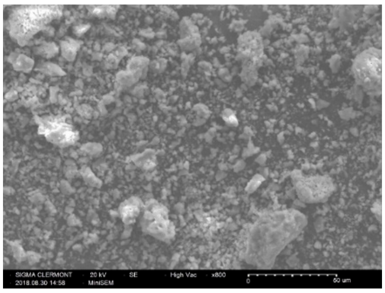
X = 20	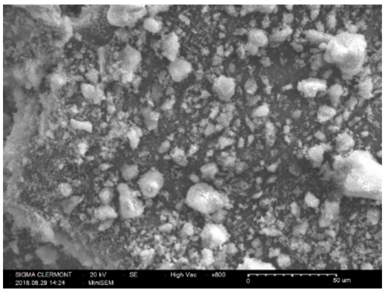	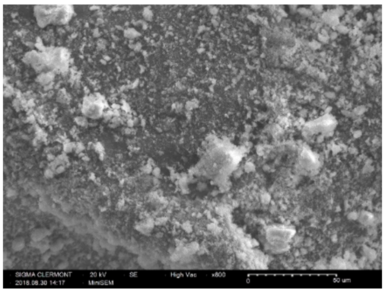	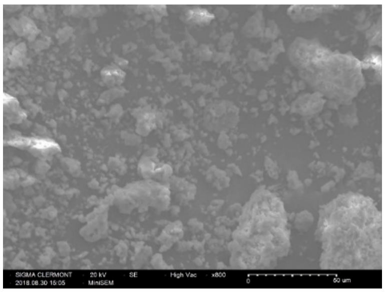

Figure S4. SEM photos for the whole *X*Cu-*T* samples with magnification 800×. Bioceramics powders were deposited on a conductive carbon adhesive coating bonded on a sample holder. Samples were coated with a thin layer of gold using a DESK V (Denton Vacuum) metallizer. SEM observations were obtained using a Hirox SH-4000 M device operating at 20 kV.

Table S1. Values of metabolic activity (%) of h-MSCs obtained after 3, 7 and 15 days of culture with different Cu-doped BCP powders. Ct+ correspond to the positive control and Ct- correspond to the negative control. Data are given as mean ± standard error from n = 9 replicates.

**Samples**	**Metabolic Activity (%)**		
	**Day 3**	**Day 7**	**Day 15**
Ct+	100 ± 12.3	100 ± 18.1	100 ± 10.1
Ct-	13.3 ± 11.1 *	21 ± 6.8 *	18.6 ± 10.7 *
00Cu-600	97.0 ± 15.9	118.5 ± 16	84.7 ± 20.8
05Cu-600	85.7 ± 19.9	104.6 ± 18.6	85.8 ± 14.3
10Cu-600	82.3 ± 13.8	98.9 ± 14.4	77.9 ± 19.4
20Cu-600	77.6 ± 22.5	100.1 ± 23.5	78.4 ± 18
00Cu-900	105.2 ± 18.1	103.4 ± 18.7	90 ± 24
05Cu-900	94.4 ± 15.2	97.5 ± 11.3	87.4 ± 21
10Cu-900	93.5 ± 13.4	92.1 ± 23.7	90.9 ± 25.5
20Cu-900	87.4 ± 18.8	99.3 ± 17.3	84.6 ± 12.1
00Cu-1200	107.6 ± 15.4	106.6 ± 20.7	82.2 ± 21.7
05Cu-1200	92.7 ± 13.2	90.8 ± 21.6	80.6 ± 22.9
10Cu-1200	85.6 ± 14.2	94.8 ± 19.6	77.8 ± 18
20Cu-1200	88.9 ± 21.5	115.5 ± 13.8	103.6 ± 8.9

* significant decrease
